# A Five-Year Overview of Bicycle Injuries in the United States Between the Years 2017 and 2021

**DOI:** 10.7759/cureus.65214

**Published:** 2024-07-23

**Authors:** Philine Endres, Nicholas W Sheets, Ian Waldrop

**Affiliations:** 1 Undergraduate Medical Education, University of California Riverside School of Medicine, Riverside, USA; 2 Trauma and Acute Care Surgery, Riverside Community Hospital, Riverside, USA

**Keywords:** bicycle, concussion, head injury, cycling, bicycle injuries

## Abstract

Introduction

This study investigates the changes in bicycle-related injury rates between 2017 and 2021. We focus specifically on changes in age demographics, and the most common diagnoses and body parts injured.

Methods

We queried the National Electronic Injury Surveillance System (NEISS) for injuries associated with bicycles from 2017 to 2021. Chi-square analysis was used to evaluate trends in injuries vs. time for the entire sample, age groups in five-year increments, and the proportion of injury types by diagnosis and body part.

Results

The highest annual injury rate (12,800 counts) occurred in 2020, coinciding with the COVID-19 safer-at-home order. Pediatric patients continue to make up the majority of injured cyclists (48% of patients are younger than 19 years), but their percentage is decreasing (zero to four years (-13%, p < 0.005), five to nine years (-17%, p < 0.005), 10-14 years (-5%, p < 0.005), 20-24 years (-16%, p < 0.005), 25-29 years (-2%, p < 0.005), and 50-54 years (-14%, p < 0.005)) and mirrored by an increase in the proportion of older injured cyclists (40-44 years (+26%, p < 0.005), 60-64 years (+44%, p < 0.005), 65-69 years (+69%, p < 0.005), and 70+ years (+57%, p < 0.005)). The past five years saw an increase in injuries associated with higher impact forces and the potential for more severe morbidity and mortality, such as internal organ injuries (+13%, p < 0.01). The incidence of concussions, however, has not changed significantly. The extremities are the most commonly injured body parts (upper and lower arm, elbow, wrist, hand, fingers, upper and lower leg, knee, ankle, foot, and toe = 47% total) and continue to increase in frequency (lower arm (+2%, p < 0.005), lower leg (+3%, p < 0.01), upper arm (+18%, p < 0.005), and hand (+11%, p < 0.05)), while facial injuries are becoming less common (-3%, p < 0.05), and head injuries have not experienced a significant change of incidence.

Conclusion

Although there was an increase in bicycle-related injuries during the COVID-19 safer-at-home order, numbers have since returned to pre-COVID-19 levels. Other changes in bicycle injury demographics and mechanisms, such as a rise in older adult cyclists and high-force mechanism injuries, however, call for a re-evaluation of preventive and treatment priorities.

## Introduction

Recreational bicycle use has been shown to have many health benefits. Simply bicycling to work regularly decreases all-cause mortality after controlling for body mass index, blood lipid levels, smoking, and blood pressure [1]. Despite these health benefits, bicycle use also poses a risk of injury. A 1992 study showed that most bicycle-related injuries were falls, followed by trauma associated with a moving vehicle [2]. The most common injury types identified were soft tissue trauma, followed by fractures, and the most common injury sites were the arms and legs [2]. A more recent study in the USA found a steady increase in age-adjusted incidence of bicycle trauma from 1998 to 2013. They also found the extremities to be the most frequently injured body part, although incidence significantly decreased, while the incidence of head and torso injuries increased over the study period [3]. There are also populations that may be at higher risk. A study evaluating bicycle-related injuries, hospital admissions, and deaths from 1997 to 2013 found rising rates of injuries and hospital admissions driven by an older patient population, even though the overall participation in cycling had not significantly changed [4]. This change in the patient population is supported by others. A study from 2006 to 2015 evaluated trauma occurring among pediatric patients and found a decreasing occurrence of bicycle-related trauma over the study years [5]. While there do appear to be demographic changes occurring among cyclists, most studies tend to focus on helmet use, as bicyclists who wear helmets are at lower risk of developing severe traumatic brain injury and at lower risk of death [6]. Additionally, studies have shown that certain populations such as teenagers are less likely to use helmets [6]. The evolving demographic changes and preventative targets, such as helmet use, necessitate a continuous need to re-evaluate bicycle injuries. The occurrence of the coronavirus disease 2019 (COVID-19) pandemic also presents a need to evaluate outdoor activities and trauma as cultural changes lead to changes in trauma mechanisms [7].

The COVID-19 pandemic encouraged people to take up new hobbies and spend more time outdoors, while the advent of electrical bicycles and other high-powered bikes has opened the sport to a wider range of participants and potentially changed bike-related injury mechanisms. One study comparing electric versus traditional pedal bikes, for example, found that people riding e-bikes tended to be older and more likely to suffer internal injuries and be hospitalized [8]. This study aims to describe bicycle injuries in the USA between 2017 and 2021. In particular, we hope to describe changes in annual injury rates, analyze age-related injury trends, and identify the most common injuries by diagnosis and body part affected. We use data on bicycle-related emergency room encounters in the United States, collected by the United States Consumer Product Safety Commission’s (CPSC) National Electronic Injury Surveillance System (NEISS).

## Materials and methods

This is a descriptive retrospective study of bicycle-related injuries in the US between the years 2017 and 2021. The NEISS is a national probability sample of approximately 100 emergency departments that gathers product-related injury data. We queried the NEISS for injuries associated with bicycles (codes 5033 and 5040) from 2017 to 2021. Data were recorded for the date of treatment, age of the patient in five-year intervals, body part injured, and diagnosis. Chi-square analysis was used to evaluate trends in injuries vs. time for the entire sample, groups in five-year increments, as well as for the proportion of injury types by diagnosis and body part. Statistical analysis was performed in IBM SPSS version 27 (IBM Corp., Armonk, NY). P-values < 0.05 were considered statistically significant. The datasets analyzed during the current study are available in the NEISS repository, CPSC NEISS On-Line Query System at CPSC.gov.

## Results

**Figure 1:**
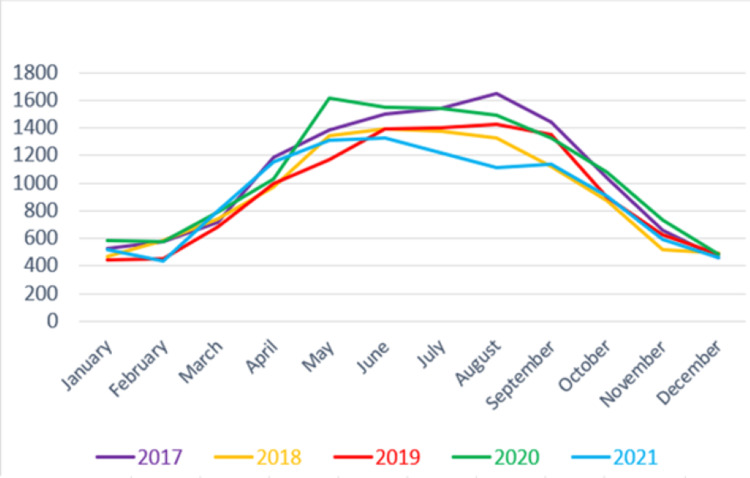
Bicycle injuries by month and year.

A total of 58,933 bicycle-related injuries were recorded in US emergency departments between the years 2017 and 2021. A comparison of the number of bicycle injuries by month and year shows that most injuries occur during the summer months (Figure 1). The average number of yearly injuries was 11,787. The study year with the most bicycle injuries was 2020 (12,800), which is a 13% increase from the previous year. The fewest yearly bicycle injuries occurred in 2021 (10,959), which is a 14% decrease from the previous year (Table 1).

**Table 1:** Trends in number of bicycle injuries from 2017 to 2021. ^a ^Indicates change from 2017 to 2021, calculated as [(value for 2021 - value for 2017)/(value for 2017)] x 100.

	2017	2018	2019	2020	2021	Total	Avg
Injuries	12,657	11,196	11,321	12,800	10,959	58,933	11,787
% Total	22	19	19	22	19	100	20
% Change^a^		-12	1	13	-14		

**Figure 2:**
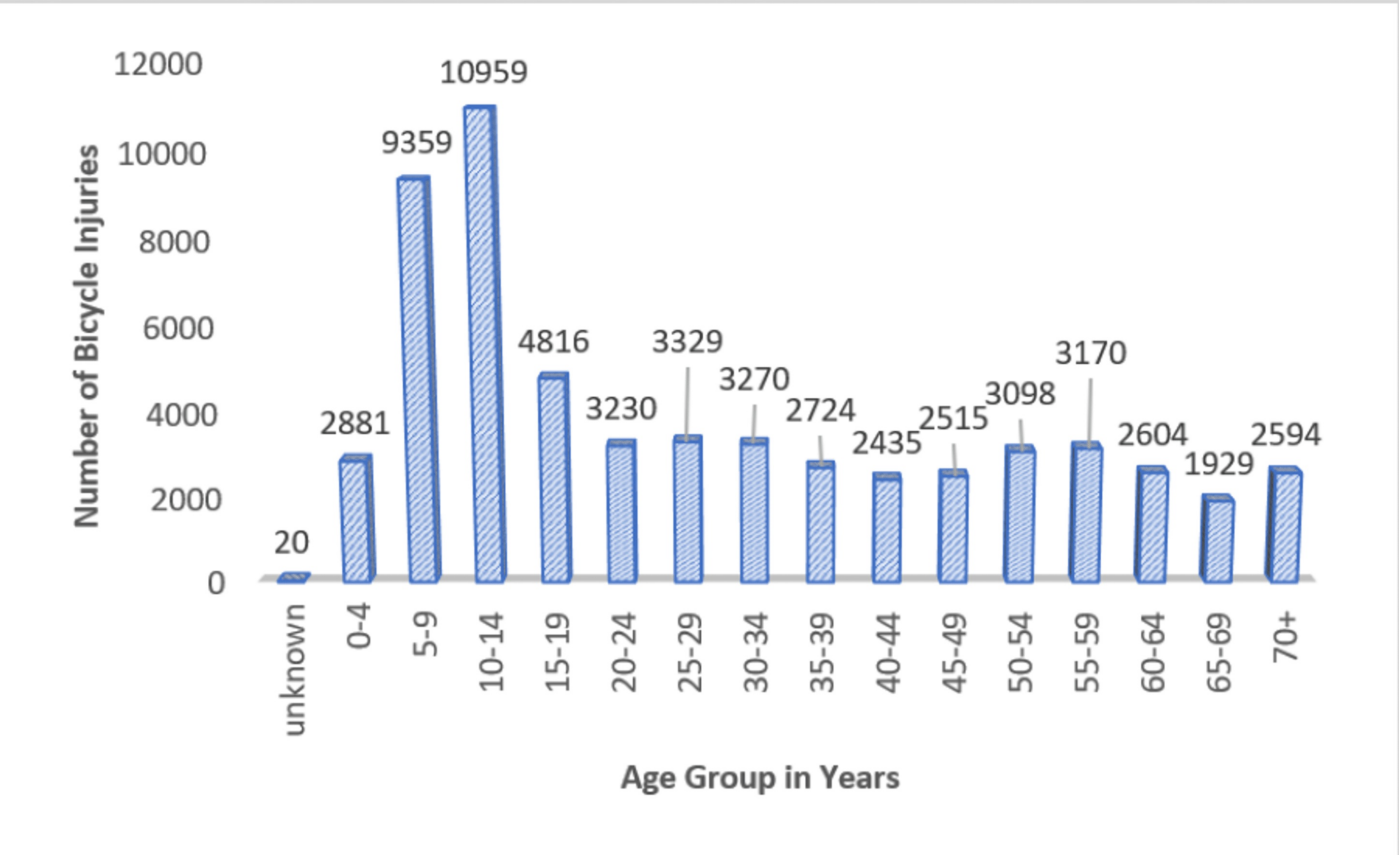
Bicycle injuries by age group from 2017 to 2021.

The average age of injury was 28.87 years old, with the most frequent age group being 10-14 years (18.6%). After the age of 15-19 years (8.2% total), injuries start to taper off with increasing age. However, there is a slight increase in injuries again among riders aged 50-54 years (5.3% total) and 55-59 years (5.4% total) (Figure 2). From 2017 to 2021, injury rates increased significantly among patients aged 40-44 years (+26%, p < 0.005), 60-64 years (+44%, p < 0.005), 65-69 years (+69%, p < 0.005), and 70+ years (+57%, p < 0.005). Age groups that experienced a significant decrease in injury rates include zero to four years (-13%, p < 0.005), five to nine years (-17%, p < 0.005), 10-14 years (-5%, p < 0.005), 20-24 years (-16%, p < 0.005), 25-29 years (-2%, p < 0.005), and 50-54 years (-14%, p < 0.005) (Table 2).

**Table 2:** Proportion of bicycle injuries by age group from 2017 to 2021. ^a ^Indicates change from 2017 to 2021, calculated as [(value for 2021 - value for 2017)/(value for 2017)] x 100. ^b ^Calculated using chi-squares for each parameter with the time period in years.

Age group (years)	2017	2018	2019	2020	2021	Total	% Change^a^	P-value^b^
Unknown	0%	0%	0%	0%	0%	0%	0%	0.5
0-4	5%	5%	4%	5%	5%	5%	-13%	<0.005
5-9	17%	16%	15%	18%	14%	16%	-17%	<0.005
10-14	19%	17%	19%	20%	18%	19%	-5%	<0.005
15-19	9%	9%	8%	8%	8%	8%	-8%	<0.05
20-24	6%	6%	6%	5%	5%	6%	-16%	<0.005
25-29	6%	6%	6%	5%	6%	6%	-2%	<0.005
30-34	6%	5%	6%	5%	6%	6%	9%	0.052
35-39	5%	5%	5%	4%	5%	5%	4%	0.541
40-44	4%	4%	4%	4%	5%	4%	26%	<0.005
45-49	4%	5%	4%	4%	4%	4%	0%	<0.05
50-54	6%	6%	5%	4%	5%	5%	-14%	<0.005
55-59	5%	6%	6%	5%	5%	5%	0%	<0.005
60-64	3%	5%	5%	5%	5%	4%	44%	<0.005
65-69	3%	3%	3%	3%	4%	3%	69%	<0.005
70+	4%	4%	4%	5%	6%	4%	57%	<0.005

The most common injury types include fractures (26% total), contusions and abrasions (19% total), lacerations (15% total), internal organ injuries (11% total), strains or sprains (8% total), and concussions (3 total%). From 2017 to 2021, injury diagnoses that increased significantly in frequency include hematomas (+27%, p < 0.01), fractures (+18%, p < 0.005), and internal organ injury (+13%, p < 0.01). Injury diagnoses that significantly decreased in frequency include contusions and abrasions (-10%, p < 0.005), lacerations (-14%, p < 0.005), dental injury (-27%, p < 0.005), and strains and sprains (-25%, p < 0.005). Of note, the incidence of concussions did not significantly change from 2017 to 2021 (Table 3).

**Table 3:** Proportion of bicycle injuries by diagnosis from 2017 to 2021. ^a^ Indicates change from 2017 to 2021, calculated as [(value for 2021 - value for 2017)/(value for 2017)] x 100. ^b^ Calculated using chi-squares for each parameter with the time period in years.

Diagnosis	2017	2018	2019	2020	2021	Total	% Change^a^	P-value^b^
Concussion	3%	3%	3%	3%	3%	3%	-15%	0.071
Contusion/abrasion	20%	21%	20%	16%	18%	19%	-10%	<0.005
Dislocation	1%	1%	1%	1%	1%	1%	17%	0.126
Fracture	24%	24%	24%	29%	28%	26%	18%	<0.005
Hematoma	1%	1%	1%	1%	1%	1%	27%	<0.01
Laceration	16%	15%	14%	15%	13%	15%	-14%	<0.005
Dental injury	1%	1%	1%	1%	1%	1%	-27%	<0.005
Internal injury	10%	11%	11%	11%	11%	11%	13%	<0.01
Strain/sprain	9%	8%	8%	6%	7%	8%	-25%	<0.005
Other	15%	16%	17%	15%	16%	16%	9%	<0.005

The most commonly injured body parts were the extremities (upper and lower arm, elbow, wrist, hand, fingers, upper and lower leg, knee, ankle, foot, and toe = 47% total), followed by the head (16% total) and face (10% total). From 2017 to 2021, body parts with a significant increase in injury rates include the lower arm (+2%, p < 0.005), lower leg (+3%, p < 0.01), upper arm (+18%, p < 0.005), and hand (+11%, p < 0.05). Body parts that experienced significantly decreased injury rates include the knee (-10%, p < 0.005), face (-3%, p < 0.05), foot (-8%, p < 0.05), and mouth (-11%, p < 0.05) (Table 4).

**Table 4:** Proportion of bicycle injuries by body part from 2017 to 2021. ^a^ Indicates change from 2017 to 2021, calculated as [(value for 2021 - value for 2017)/(value for 2017)] x 100. ^b^ Calculated using chi-squares for each parameter with the time period in years.

Body part	2017	2018	2019	2020	2021	Total	% Change^a^	P-value^b^
Shoulder	7%	7%	8%	7%	8%	7%	8%	0.509
Trunk, upper	7%	7%	7%	6%	7%	7%	4%	0.051
Elbow	5%	5%	5%	5%	5%	5%	-12	0.057
Arm, lower	6%	5%	6%	7%	6%	6%	2%	<0.005
Knee	7%	7%	7%	6%	6%	7%	-10%	<0.005
Leg, lower	6%	5%	6%	5%	6%	6%	3%	<0.01
Ankle	4%	4%	4%	4%	4%	4%	0%	0.879
Pubic region	1%	1%	1%	2%	1%	1%	0%	<0.05
Head	16%	16%	16%	16%	16%	16%	1%	0.909
Face	10%	10%	10%	11%	10%	10%	-3%	<0.05
Eyeball	0%	0%	0%	0%	0%	0%	33%	0.395
Trunk, lower	6%	7%	6%	6%	6%	6%	2%	0.279
Arm, upper	1%	1%	1%	2%	1%	1%	18%	<0.005
Leg, upper	2%	2%	2%	2%	2%	2%	5%	0.595
Hand	3%	3%	3%	3%	3%	3%	11%	<0.05
Foot	2%	2%	2%	2%	2%	2%	-8%	<0.05
Mouth	3%	2%	3%	3%	2%	3%	-11%	<0.05
Neck	2%	1%	2%	1%	2%	1%	0%	<0.05
Finger	4%	4%	3%	3%	3%	4%	-11%	0.102
Toe	1%	1%	1%	1%	1%	1%	0%	0.052
Other	2%	2%	2%	2%	2%	2%	22%	0.077

## Discussion

Bicycle injuries peak in the summer months when weather conditions are most conducive to riding bicycles outdoors [9]. During 2020, marked by the COVID-19 safer-at-home order, bicycle-related injuries were higher than in the previous year. This is likely due to the closure of gyms, and social distancing efforts encouraging solo outdoor exercise [10,11]. Interestingly, there was a great decrease in bicycle-related injuries during the summer of 2021. This may be due to it being the hottest summer recorded in 126 years in the contiguous US, discouraging cyclists from exercising outdoors [12]. It could also be related to gyms reopening in the spring of that year and many people switching from cycling back to their indoor exercise routines [10].

The predominance of bicycle-associated injuries among children and adolescents is likely attributable to inexperienced and often un-helmeted riders in this group [6]. Injury rates fall as riders gain experience and ride bikes less often. The resurgence of injury rates among older riders may be associated with most e-bike riders being over 50 years old, combining higher riding speeds with more injury-prone bodies [13]. Pediatric patients still constitute the majority of injured cyclists but make up a decreasing percentage of all casualties while the proportion of older injured cyclists is increasing from year to year. This confirms the continuation of an advanced age trend among bicycle injuries identified in earlier research [4,5].

Fractures and soft tissue injuries continue to be the most frequent injuries sustained from bicycle accidents. This seems to be a continuing trend since the 1990s [2]. However, less severe soft tissue injuries such as contusions, abrasions, and lacerations have decreased in incidence during this study. Strains and sprains have also declined, while more severe injury types such as fractures and internal organ injuries have increased in frequency. This increase in injuries associated with higher force impacts may reflect advances in bicycle technology, such as e-bikes and downhill mountain bikes [13]. It is also interesting to note that the incidence of concussions, though heavily featured in research and policy, has not changed significantly in the past five years.

The most commonly injured body parts were the extremities, a finding that has continued to be consistent since the 1990s [2]. However, the incidence of extremity trauma has been decreasing, contrary to the findings of earlier studies [3]. The head and face were the next most likely to be injured, with the head being the single most injured body part in every year of the study. However, injury rates for the head and face have not been increasing, unlike previous predictions [3]. In fact, the incidence of maxillofacial trauma has been decreasing, possibly due to the widespread use of full-face helmets and other protective gear. The incidence of head trauma has not changed significantly, despite being the center of public and academic attention.

This study has some limitations associated with the NEISS database. Most importantly, the incidence of bicycle injuries may be underestimated. Injuries that involved more than one product, for example, bicycles and stairs, may be listed under the alternate category. Those who did not seek medical attention at a hospital with an ED participating in the database were also not included, although national estimates aim to account for these missing data points. Additionally, a few participating emergency departments did not report data on certain variables, including patient age. Fortunately, the number of patients falling into the age category “unknown” made up less than 1% of the total sample. Similar to injuries involving multiple products, injuries causing polytrauma or affecting multiple body parts were listed only under the primary diagnosis. Therefore, the incidence of individual diagnoses or body parts injured may be underestimated as well. All of these factors may limit the generalizability of this database, and since some of the pandemic directives mentioned in this study were specific to the United States alone, these findings may not be applicable to other countries.

## Conclusions

Although there was an increase in bicycle-related injuries during the COVID-19 safer-at-home order, numbers have since returned to pre-COVID levels. A trend that continues to endure, however, is the increasing incidence of cycling injuries among patients over the age of 60 years, associated with a decreasing incidence of injuries among pediatric cyclists. Furthermore, injuries associated with higher impact forces and more potential for significant mortality and morbidity are increasing as well. The higher speeds and more challenging terrain that can be attempted on modern bicycles, along with an older population of riders, make for a dangerous injury profile. Past research and policy have focused tirelessly on reducing the impact of concussions and head injuries among cyclists. This study, however, finds no significant change in the incidence of these injuries, indicating that current interventions may not be sufficient. However, it should be noted that this is happening in the setting of increased speed and widening applicability of all-terrain bicycles. Furthermore, physicians need to be aware of new demographic and injury trends to identify and counsel riders at risk, triage and treat injured cyclists, and support safer riding campaigns and legislature.
